# Post-Thaw Sperm Quality and Functionality in the Autochthonous Pig Breed Gochu Asturcelta

**DOI:** 10.3390/ani11071885

**Published:** 2021-06-24

**Authors:** José Néstor Caamaño, Carolina Tamargo, Inmaculada Parrilla, Felipe Martínez-Pastor, Lorena Padilla, Amer Salman, Carmen Fueyo, Ángel Fernández, María José Merino, Tania Iglesias, Carlos Olegario Hidalgo

**Affiliations:** 1 Department of Animal Selection and Reproduction, Regional Service for Agrifood Research and Development (SERIDA), 33394 Gijon, Spain; ctamargo@serida.org (C.T.); picarol@gmail.com (C.F.); angelfg@serida.org (Á.F.); mjosemh3@gmail.com (M.J.M.); cohidalgo@serida.org (C.O.H.); 2 Department of Animal Medicine and Surgery, Faculty of Veterinary Medicine, University of Murcia, 30071 Murcia, Spain; parrilla@um.es (I.P.); lorenaconcepcion.padilla@um.es (L.P.); 3 INDEGSAL, Universidad de León, 24071 León, Spain; felipe.martinez@unileon.es (F.M.-P.); asalma00@estudiantes.unileon.es (A.S.); 4 Molecular Biology (Cell Biology), Universidad de León, 24071 León, Spain; 5 Unidad de Consultoría Estadística, Universidad de Oviedo, 33203 Gijón, Spain; iglesiasctania@uniovi.es

**Keywords:** pig, autochthonous breeds, sperm cryopreservation, sperm quality, seasonality

## Abstract

**Simple Summary:**

Genetic resource banks were created to preserve the genetic material of endangered, rare, valuable individuals or genetically relevant breeds. Sperm cryopreservation is a practical and widespread strategy to preserve these genetic materials. This study aimed to characterize the frozen-thawed sperm of the native pig breed Gochu Asturcelta, considering the effects of boar age and season of semen collection on post-thaw sperm quality. We found that the boar age did not have a significant effect on the sperm parameters assessed. However, the season significantly affected many of these parameters (motility, viability, acrosomal status, mitochondrial activity). In general, sperm samples collected in spring and summer showed higher quality post-thawing, the lowest in winter. Our findings demonstrated that the post-thawing sperm quality of Gochu Asturcelta was in the range of results for commercial breeds, bringing a good prospect for the use of assisted reproductive technologies in this local breed.

**Abstract:**

Genetic resource banks (GRB) preserve the genetic material of endangered, valuable individuals or genetically relevant breeds. Semen cryopreservation is a crucial technique to reach these goals. Thus, we aimed to assess the sperm parameters of semen doses from the native pig breed Gochu Asturcelta stored at the GRB of Principado de Asturias (GRB-PA, Gijón, Spain), focusing on intrinsic and extrinsic (boar, season) factors. Two straws per boar (*n* = 18, 8–71 months of age) were thawed, pooled, and assessed after 30 and 150 min at 37 °C by CASA (computer-assisted sperm analysis system; motility and kinematic parameters) and flow cytometry (viability, acrosomal status, mitochondrial activity, apoptosis, reactive oxygen species, and chromatin status). The effects of age, incubation, and season on post-thawing quality were determined using linear mixed-effects models. Parameters were on the range for commercial boar breeds, with chromatin status (SCSA: fragmentation and immaturity) being excellent. Incubation decreased sperm quality and functionality. The boar age did not have a significant effect (*p* > 0.05), but the between-boar variability was significant (*p* < 0.001). The season significantly affected many parameters (motility, kinematics, viability, acrosomal status, mitochondrial activity), especially after 150 min of incubation. In general, samples collected in spring and summer showed higher quality post-thawing, the lowest in winter. In conclusion, the sperm doses from the Gochu Asturcelta breed stored at the GRB-PA showed excellent chromatin status and acceptable characteristics after thawing. Therefore, boar and seasonal variability in this autochthonous breed could be relevant for cryobank management.

## 1. Introduction

For some domestic species, extinction has been due to the intensive selection of a few breeds imposed by management techniques and market demands [1]. In general, native breeds have limited profitability, causing local producers and breeders to lose interest and replace them with commercial ones [2]. However, the need to preserve genetic variability in domestic livestock has been recognized for many years [3]. These breeds present characteristics not found in the more productive and selected commercial breeds, such as rusticity, adaptation to particular environments, good maternal qualities, longevity, and disease resistance of great interest in a world facing environmental challenges [2,4,5,6]. The preservation of genetic pools is critical for animal conservation because it prevents the loss of genetic diversity, an essential factor for protecting populations during unforeseen situations. Therefore, the challenges forced by global climate change have triggered a renewed interest in them.

These breeds are also cultural and historically valuable, meriting for preservation for future generations. Also, they contribute to preserving the rural societies that rear them [5,7,8,9]. This is the case of the pig breed Gochu Asturcelta, a native of Northern Spain. It was once typical of the mountainous region of Asturias, but it was pushed to the brink of extinction [10]. However, the efforts of the breeders’ association (Asociación de Criadores de Gochu Asturcelta-ACGA) together with the local government (Gobierno del Principado de Asturias) and the regional centre for livestock resources preservation (SERIDA) have enabled its recovery, increasing the interest to rear this autochthonous and traditional breed [10,11].

Genetic resource banks were created to preserve the genetic material of endangered, rare, valuable individuals or genetically relevant breeds to support conservation and diffusion programs [12]. In this regard, sperm cryopreservation is a practical and widespread strategy [13]. Moreover, the storage of boar semen in frozen form is highly desirable to maintain germplasm, representing critical economic traits, preserving genetic diversity, providing more efficient breeding, and serving as a reserve in case of germplasm loss [14]. Although sperm cryopreservation in swine is well documented, it is still far from being practical in commercial breeds [15,16,17,18,19]. Reduced fertility with frozen-thawed semen has been associated with cell damage altering motility, viability, capacitation, DNA, membrane structure, and acrosomal integrity, and increased reactive oxygen species and lipid peroxidation [20,21,22].

Therefore, applying these techniques in autochthonous breeds such as the Gochu Asturcelta could present further challenges due to the lack of information on the breed’s physiology. This problem is shared with most indigenous breeds and hampers its recovery by threatening germplasm banking’s viability [8,9,23]. Furthermore, the Gochu Asturcelta pig breed, like many other autochthonous breeds, has not been characterized regarding the post-thawing quality of the frozen semen, and sperm parameters have not been described in detail yet. Since standard cryopreservation protocols may not be entirely suitable for native breeds, the need to study in detail the stock of frozen sperm doses of Gochu Asturcelta in the GRB of the Principado de Asturias is mandatory. Moreover, due to its rusticity, we hypothesize that this breed could show seasonal variability in the semen characteristics, affecting the post-thawing quality. This has been reported in commercial breeds [24,25], and it could be more evident in autochthonous ones.

Therefore, the objective of this study was to characterize the frozen-thawed sperm of the pig breed Gochu Asturcelta, considering the effects of boar age and season of semen collection on post-thaw sperm quality. In addition, the results of this study could be of interest for the preservation of other local, non-commercial pig breeds.

## 2. Material and Methods

### 2.1. Reagents

All chemicals were purchased from Sigma-Aldrich (Merck KGaA, Darmstadt, Germany) unless otherwise indicated.

### 2.2. Genetic Resource Bank Location and Climate

Boars were housed in a swine facility at the Animal Biotechnology Center, SERIDA, Gijón, Spain, where the GRB for Endangered Domestic Native Animal Species of Principado de Asturias (GRB-PA) is located (43°32′8.63″ N, 5°39′41.47″ W). The general climate in Asturias is oceanic, with abundant rainfall throughout the year and mild temperatures in both winter and summer. The average of the warmest month does not exceed 20 °C, with a reduced thermal amplitude (between 8 and 15 °C). There are no dry months in summer. The warmest temperatures occur on the coast between Gijón and Villaviciosa (Asturias), around 19 °C (from AEMET, Spanish Meteorological Agency, http://www.aemet.es, accessed on 5 June 2021). Daily temperature and rainfall measurements for the association analysis with sperm quality measurements were obtained within the R statistical environment v. 3.6.3 from AEMET OpenData with the meteoland package [26] and daylength with the geosphere package [27]. Meteorological and daylength statistics were obtained for seven weeks before collection day and averaged.

### 2.3. Boars, Semen Collection and Cryopreservation Procedure

The semen doses assessed in this study were obtained from 18 Gochu Asturcelta boars, ranging from 8 to 71 months of age (replicate collections except for two males). The cryobank contained 54 different freezing lots: 21 collected in spring, 13 in summer, 12 in autumn, and 8 in winter. For semen collection, the sperm-rich fraction of the ejaculate was collected using the gloved-hand method (the end of the penis is grasped with a gloved hand and exerts pressure on it to stimulate ejaculation) [28], extended (1:1, vol/vol) in Beltsville Thawing Solution (BTS) [29]. Fresh semen was assessed after collection for concentration (Accucell photometer, IMV, L’Aigle, France) and motility using a phase-contrast microscope. Only ejaculates with more than 200 × 10^6^ spermatozoa/mL, 75% sperm motility, and 80% spermatozoa with normal morphology were accepted for freezing. At least two ejaculates per boar, collected on different days, were frozen following the procedure described by Carvajal et al. [28]. Briefly, extended sperm-rich fractions were slowly cooled to 17 °C for 240 min. The extended sperm suspension was centrifuged at 2400× *g* for 3 min in a refrigerated centrifuge at this temperature. After centrifugation, the pellets were diluted to a concentration of 1500 × 10^6^ mL^−1^ with lactose-egg yolk (LEY) extender, composed of 80% (*v/v*) β-Lactose solution (310 mM in distilled water), 20% (*v/v*) egg yolk, and 100 mg/mL kanamycin sulfate. After further cooling to 5 °C for 120 min, diluted spermatozoa were resuspended to a final concentration of 10^9^ mL^−1^ with LEY glycerol-Orvus-ES-Paste (LEYGO) extender, consisting of 92.5% (*v/v*) LEY, 6% (*v/v*) glycerol and 1.5% (*v/v*) Equex STM. French straws (0.5 mL) were filled with extended semen. Straws were horizontally frozen using a programmable cell freezer (MiniDigitcool 1400, IMV): From 5 °C to −5 °C at a rate of 6 °C/min, from −5 °C to −80 °C at a rate of 40 °C/min, held for 30 s at −80 °C, and then cooled from 70 °C/min to −150 °C. Then, the straws were plunged into liquid nitrogen and stored in a liquid nitrogen container [28]. The semen doses were stored in the GRB-PA at SERIDA until used in this study. 

### 2.4. Assessment of Post-Thaw Sperm Parameters

Two straws per boar and ejaculate were thawed in a water bath (37 °C, 20 s), pooled, and the content was extended 1:1 (*v/v*) in BTS. Frozen-thawed diluted sperm samples were assessed after 30 and 150 min of incubation at 37 °C [28,30]. Sperm were evaluated for motility (total and progressive motility and kinematic parameters), viability, intact acrosome membrane, mitochondria membrane potential, intracellular reactive oxygen species (ROS) generation, apoptosis, and chromatin status. The flow cytometry analyses were performed using a FACSCanto II cytometer (Becton Dickinson Co., Franklin Lakes, NJ, USA), except for the chromatin status, assessed with a FACScalibur cytometer (Becton Dickinson; description at 2.4.6). The FACSCanto II was equipped with a standard configuration, using the lasers and filters for the fluorescent probes used (techniques described from 2.4.2 to 2.4.5) as follows: The fluorescence spectrum of Hoechst 33,342 (blue) was detected using a 450/50 nm bandpass (BP) filter, the red fluorescence spectra of propidium iodide were detected using a 670 nm long-pass filter (LP), and Mitotracker deep red was detected using a 660/20 BP filter. The green fluorescence emission for PNA-FITC, CH-H_2_DCFDA, and Annexin-V was detected using a 530/30 BP filter. The FACSCanto FCS files were analyzed using BD FACS Diva™ Software v.6.1.3 (Becton Dickinson Co., Franklin Lakes, NJ, USA). The gating and analysis strategy, including representative cytograms and histograms, as shown in the Supplementary Material, Figure S1.

#### 2.4.1. Sperm Motility

Sperm motility was objectively evaluated using a computer-assisted sperm analysis system (CASA) (ISAS; Proiser R + D, Paterna, Spain) following the procedure described by Li et al. [31]. Briefly, 5 µL aliquots of thawed sperm samples (at 20–30 × 10^6^ sperm/mL in BTS) were placed in a pre-warmed (38 °C) Makler counting chamber (Sefi Medical Instruments, Haifa, Israel), and 3–4 fields (at least 500 spermatozoa per sample) were analyzed at 100× using an UB200i microscope equipped with an ISAS 782M video camera (both from Proiser R + D SL, Paterna, Spain). Each sperm trajectory was recorded, and each field was visually assessed to eliminate possible debris and minimize the risk of including unclear sperm tracks in the analysis. Sperm motility was recorded as the percentage of total motile spermatozoa (average path velocity ≥20 μm/s) and sperm showing rapid and progressive movement (straightness of the average path ≥40%). In addition, kinetic parameters were also recorded, as the curvilinear velocity (VCL, µm/s), straight-line velocity (VSL, µm/s), average path velocity (VAP, µm/s), linearity of sperm movement (LIN, %), straightness of the average path (STR, %), wobble coefficient (WOB, %), the amplitude of lateral head displacement (ALH, µm), sperm dance (DNC, ALH × VCL), sperm mean dance (DNCm, ALH × VCL/VSL) and beat cross frequency (BCF, Hz).

#### 2.4.2. Viability and Acrosomal Status

Sperm viability and acrosomal status were evaluated as plasma and acrosome membrane integrity using a triple-fluorescence procedure by flow cytometry [32]. One hundred microliters of each sample (30 × 10^6^ mL^−1^ in BTS) were stained with 3 µL of Hoechst 33,342 (H-42) (0.05 mg/mL in PBS), 2 µL of propidium iodide (PI; Invitrogen™, Thermo Fisher Scientific, Waltham, MA, USA; 0.5 mg/mL in PBS) and 2 µL of fluorescein isothiocyanate-conjugated peanut agglutinin (PNA-FITC) (200 μg/mL in PBS). Samples were incubated in the dark at 37 °C for 10 min. Four hundred microliters of PBS were added to each sample before cytometry analysis. A total of 10,000 H-42 positive events were recorded for each sample, and the viable sperm population (PI negative) showing non-reacted (PNA-FITC negative) or reacted (PNA-FITC positive) acrosome were recorded.

#### 2.4.3. Mitochondrial Membrane Potential

Mitochondrial membrane potential was evaluated in sperm samples (100 µL containing 3 × 10^6^ sperm) incubated (38 °C for 15 min in the dark) with 3 µL of H-42 (0.05 mg/mL in PBS), 2 µL of PI (0.5 mg/mL in PBS), and 0.5 µL of Mitotracker Deep Red 633 (Mitotracker, 20 µM in PBS of a stock solution of 1 mM in DMSO) as it was described by Perez-Patiño et al. [33]. The spermatozoa positive to H-42 and Mitotracker and negative to PI were recorded as viable with high mitochondrial membrane potential. Therefore, the ratio of Mitotracker positive spermatozoa within the viable population was used in this study.

#### 2.4.4. Reactive Oxygen Species (ROS)

The intracellular generation of ROS was measured in terms of hydrogen peroxide (H_2_O_2_) generation. Intracellular H_2_O_2_ generation in viable sperm was measured using 5(and-6)chloromethyl-2’, 7’-dichlorodihydrofluorescein diacetate, acetyl ester (CM-H_2_DCFDA; Invitrogen™, Thermo Fisher Scientific, Waltham, MA, USA) following Guthrie and Welch [34]. Briefly, 50 µL of each sperm sample (30 × 10^6^ mL^−1^) was extended in 950 µL of PBS containing 1.5 µL of H-42 (0.05 mg/mL in PBS), 1 µL of PI (0.5 mg/mL in PBS) and 1 µL of CM-H_2_DCFDA (1 mM in DMSO). A similar sperm sample, mixed with 1 µL of tert-butyl hydroperoxide (TBH) solution (70% in distilled water), was used as a positive control. The sperm samples were incubated in the dark at 37 °C for 30 min before cytometric analysis. Spermatozoa negative for PI and positive for dichlorofluorescein were considered viable with high intracellular H_2_O_2_ generation [34]. Therefore, the ratio of ROS-positive spermatozoa within the viable population was used in this study.

#### 2.4.5. Apoptosis

The early apoptosis events were evaluated using the fluorescent probes Annexin V-FITC (Invitrogen™, Thermo Fisher Scientific, Waltham, MA, USA) as described by Li et al. [35]. Briefly, 45 µL of sample (20 × 10^6^ mL^−1^) were mixed with 180 µL Annexin-V binding buffer and 5 µL H-42 (0.05 mg/mL in PBS), 1 µL PI (0.5 mg/mL in PBS) and 3 µL Annexin V-FITC. After incubation in the dark at room temperature for 15 min, 200 µL Annexin-V binding buffer was added just before flow cytometry analysis. A total of 10,000 events were analyzed. Viable spermatozoa (H-42 positive and PI negative) showing early signs of apoptosis (Annexin V-FITC positive) were recorded.

#### 2.4.6. Sperm Chromatin Status (Sperm Chromatin Structure Assay, SCSA)

Chromatin stability was assessed by SCSA [36]. The technique is based on the denaturalization of broken DNA and on the properties of Acridine orange (AO), whose fluorescence shifts from green (double-stranded, dsDNA) to red (single-stranded, ssDNA) depending on the degree of DNA denaturation. Samples were diluted in TNE buffer (0.01 M Tris-HCl, 0.15 M NaCl, and 1 mM disodium EDTA, pH 7.4) to 2 × 10^6^ mL^−1^ and stored at −80 °C. For analysis, samples were thawed on crushed ice. A 200-µL aliquot of the sample was pipetted into a flow cytometry tube and immediately mixed with 0.4 ml acid-detergent solution (0.08 M HCl, 0.15 M NaCl, and 0.1% Triton X-100, pH 1.2). After 30 s, 1.2 mL of staining solution (6 µg/mL AO (Polysciences, Inc., Warrington, PA, USA) in 0.1 M citric acid, 0.2 M Na_2_HPO_4_, 1 mM disodium EDTA and 0.15 M NaCl, pH 6.0) was added to the tube. The tube was kept on ice for 3 min before analysis. A FACScalibur flow cytometer was used for analysis with acquisition software CellQuest version 3.1 (Becton Dickinson). At least 5000 spermatozoa were analyzed per sample, exciting the AO with an Ar-ion laser at 488 nm and using a 530/30 filter for the green fluorescence of dsDNA-bound AO and a 650 long-pass filter for the red fluorescence of ssDNA-bound AO. Data were saved in flow cytometry standard (FCS) version 2 files. The DNA fragmentation index (DFI) was calculated for each sperm as the red fluorescence ratio to total (red + green) fluorescence. From the %DFI, the standard deviation of DFI (SD-DFI) and the percentage of spermatozoa with a high fragmentation index (DFI > 25%; %hDFI) were determined. Chromatin immaturity was estimated as %HDS, defined as the proportion of events with high green fluorescence.

### 2.5. Statistical Analysis

Data were analyzed in the R statistical environment version 3.6.3 [37] using linear mixed-effects models (LME, lmerTest package [38], and multcomp [39]). Incubation time, boar age (in months), and season of semen collection were included as fixed factors, with boar as the grouping factor in the random part of the models. Post hoc multiple comparison analyses corrected by Holm-Bonferroni were used. For improving the interpretation of the season effects, we performed another LME, including the meteorological variables mean day temperature and precipitation and the day length. The level of significance was set at *p* < 0.05.

## 3. Results

**Figure 1:**
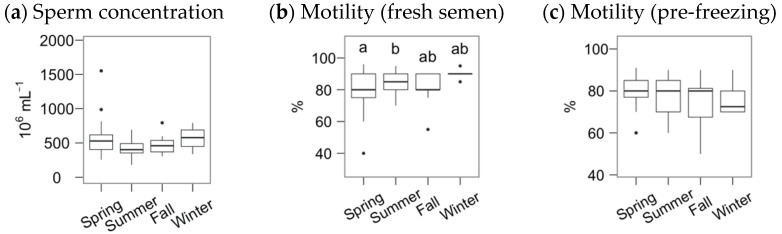
Fresh and pre-freezing statistics for Gochu Asturcelta semen, with sperm concentration (**a**) and subjective motility (**b**) of the freshly collected semen, and subjective motility of the pre-freezing, cooled and extended, semen (**c**). Box plots show 1st and 3rd quartiles of the distribution (box limits), median (inner line), upper and lower hinges of the distribution (vertical lines, extreme observations within 1.5 times the interquartile limits), and outliers (dots, observations outside the upper/lower hinges). Seasons with different letters (within each analysis time) differ significantly (*p* < 0.05).

The average sperm concentration and subjective motility for the Gochu Asturcelta fresh semen samples were (median and interquartile range, IRQ) 470 (378, 598) × 10^6^ mL^−1^ and 85% (80%, 90%), respectively, with pre-freezing motility of 80% (70%, 85%). The boar age did not significantly influence these variables. However, the season significantly affected the motility of the fresh semen (Figure 1), being slightly higher in the summer compared to the spring.

Sperm characteristics for cryopreserved spermatozoa of the Gochu Asturcelta breed showed acceptable characteristics after thawing. The overall data distributions for the sperm kinematic parameters, flow cytometry, and chromatin status for all the frozen samples are shown in Table 1 and Table 2 (post-thawing) and Table 3 and Table 4 (after 150 min of incubation).

All samples post-thawing showed total motility above 30% and viability above 50%, with half of the samples above 30% of progressive motility. Flow cytometry analyses supported the acceptable status of the thawed samples, with a relatively high proportion of viable spermatozoa, and in general, with low proportions of spermatozoa with apoptotic features or high cytoplasmic ROS. 

The values for the sperm chromatin status parameters were mainly below the threshold for impaired fertility in pigs (%DFI: 6%; %HDS: 15%). All samples yielded %DFI below 3%, and all, except two samples, were below 15% for %HDS.

The sperm quality noticeably decreased after the incubation (*p* < 0.001) as expected, except for the linearity parameters LIN, STR, and WOB, BCF, the ROS ratio, and chromatin parameters SD-DFI, %DFI and %HDS.

The analysis of the statistical models showed no effect of boar age (*p* > 0.05). However, despite this lack of effect, the intrinsic boar variability (random-effects analysis) was relevant and significant for all variables (*p* < 0.001, except for SD-DFI and %HDS with *p* < 0.05).

**Figure 2:**
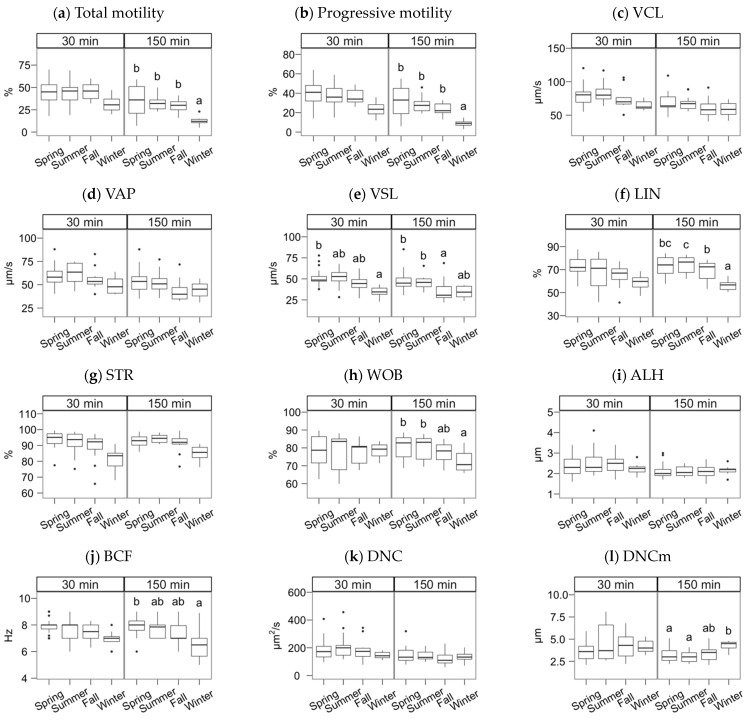
CASA features of Gochu Asturcelta spermatozoa collected and cryopreserved at different moments along the year and after thawing (30 min) and after 150 min of incubation at 37 °C. (**a**) Total motility; (**b**) Progressive motility; (**c**) Curvilinear velocity; (**d**) Average-path velocity; (**e**) Straight-path velocity; (**f**) Linearity (ratio of VSL/VCL); (**g**) Straightness (VSL/VAP); (**h**) Wobble (VAP/VCL; (**i**) The amplitude of the lateral displacement of the sperm head; (**j**) Frequency of the flagellar beat; (**k**) Sperm Dance; (**l**) Sperm mean Dance. Box plots show 1st and 3rd quartiles of the distribution (box limits), median (inner line), upper and lower hinges of the distribution (vertical lines, extreme observations within 1.5 times the interquartile limits), and outliers (dots, observations outside the upper/lower hinges). Seasons with different letters (within each analysis time) differ significantly (*p* < 0.05).

**Figure 3:**
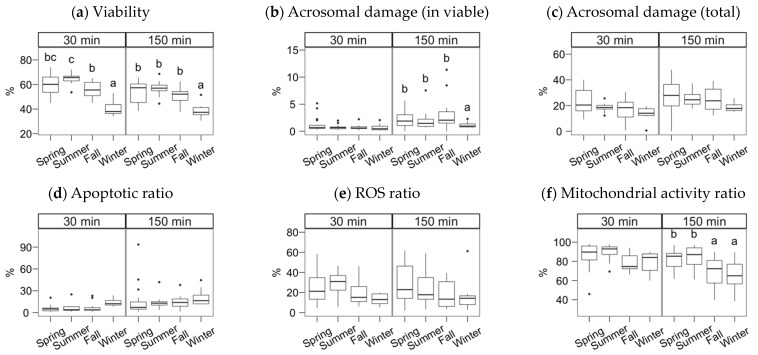
Sperm physiological parameters as assessed by flow cytometry. Gochu Asturcelta spermatozoa collected and cryopreserved at different moments along the year and after thawing (30 min) and after 150 min of incubation at 37 °C. (**a**) Sperm viability as PI^–^; (**b**) Acrosomal damage as %PNA^+^ in PI^−^; (**c**) Total sperm with damaged acrosomes (%PNA^+^); (**d**) Viable sperm with apoptotic features as Annexin^+^ in PI^−^; (**e**) Viable sperm producing ROS as H_2_DCFDA^+^ in PI^−^; (**f**) Viable sperm with active mitochondria as Mitotracker^+^ in PI^−^. Box plots show 1st and 3rd quartiles of the distribution (box limits), median (inner line), upper and lower hinges of the distribution (vertical lines, extreme observations within 1.5 times the interquartile limits), and outliers (dots, observations outside the upper/lower hinges). Seasons with different letters (within each analysis time) differ significantly (*p* < 0.05).

**Figure 4:**
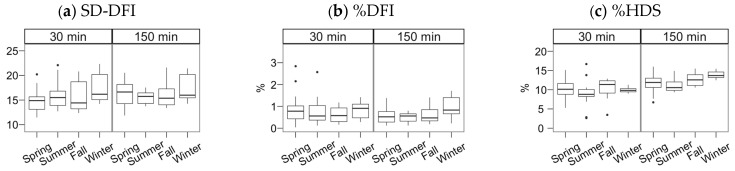
Chromatin status (SCSA technique) for Gochu Asturcelta spermatozoa collected and cryopreserved at different moments along the year and after thawing (30 min) and after 150 min of incubation at 37 °C. (**a**) SD-DFI: Standard deviation of the DNA fragmentation index (DFI); (**b**) %DFI: proportion of spermatozoa with high DFI (damaged DNA); (**c**) %HDS: proportion of spermatozoa with immature or decondensed chromatin. Box plots show 1st and 3rd quartiles of the distribution (box limits), median (inner line), upper and lower hinges of the distribution (vertical lines, extreme observations within 1.5 times the interquartile limits), and outliers (dots, observations outside the upper/lower hinges). Neither the season nor the incubation significantly affected the studied parameters.

The semen collection and freezing season exerted a significant effect but, in most cases, only noticeable after incubating the spermatozoa (Figure 2, Figure 3 and Figure 4; multiple comparisons between seasons detailed in Supplementary Materials File S1). In general, samples collected in spring and summer showed higher quality post-thawing, the lowest in winter. This quality decrease was significant 30 min after thawing for VSL (*p* < 0.05) and viability (*p* < 0.001). 

After the incubation (150 min), the season effect was significant for total and progressive motility (Figure 2a,b), VSL and linearity parameters (Figure 2e–h), BCF (Figure 2j), viability, the ratio of viable spermatozoa with reacted acrosomes (Figure 3a,b), and the ratio of viable spermatozoa with active mitochondria (Figure 3f). Winter was significantly lower for total and progressive motility than the other three seasons (*p* < 0.001 for spring, *p* < 0.01 for summer and autumn). For the kinematic variables, the distribution was more heterogeneous, but winter always yielding lower values, except for DNCm, with higher values. The trend for viability at 150 min was similar to total motility (*p* < 0.001 for spring and summer, *p* < 0.01 for autumn), but for the mitochondrial activity ratio, there was an apparent dichotomy between higher spring-summer values and autumn-winter (*p* < 0.05). Interestingly, the reacted acrosomes ratio decreased in the winter, with *p* < 0.05.

A model including precipitation, mean daily temperature, and photoperiod (daylight hours) was included to clarify further the possible mechanism of the season effect (exact *p* values for the effects shown in Supplementary Materials File S2). The meteorological variables did not affect sperm quality. Only the sperm concentration and motility in the fresh semen were affected (*p* = 0.004) by the daily mean temperature (negatively for concentration and positive motility). However, the daylight seemed to influence some sperm parameters post-thawing. At 30 min post-thawing, the VCL (*p* = 0.033) and viability (*p* = 0.001) were positively affected by daylight hours. At 150 min, we found this effect for the kinematic parameters VSL, VAP, LIN, STR, and WOB, positively affected by daylight (*p* < 0.05). Viability (*p* = 0.013) and mitochondrial activity (0.005) were also positively affected. Interestingly, the day length negatively affected the chromatin condensation as %HDS (*p* = 0.003).

## 4. Discussion

This study presents an example of the application of sperm cryopreservation to preserve a local, endangered breed, the Gochu Asturcelta, by analyzing the semen samples stored at GRB-PA at SERIDA. One of the most relevant findings in our study is the overall characterization, for the first time, of the post-thawing sperm quality of this breed. Although some studies reported no differences in sperm quality among pig breeds [40], recent studies have highlighted the importance of pig genetics and the breed on the characteristics of the ejaculate [41,42,43], and possibly on the cryopreservation aptitude [44]. Therefore, the detailed characterization of sperm quality guarantees the future application of the sperm doses stored in the germplasm bank. This characterization is even more critical considering the sensitivity of the boar sperm to freezing damage compared to other domestic species, mainly due to its low cholesterol/phospholipid ratio [45,46], hampering the outcomes of sperm cryopreservation.

This study reports a post-thawing sperm quality within the range of commercial breeds, although average values for some parameters, such as total and progressive motility, are slightly lower [15,25,31]. This potentially lower post-thawing quality could be explained because of the recent history of the breed. The Gochu Asturcelta breed was at the brink of extinction, and its recovery has been achieved from a population of few animals. Most of the efforts have been directed to increase the population size while reducing inbreeding. Therefore, other features, such as fertility, have been neglected, which could have influenced some of the results obtained in this study.

The parameters assessed by flow cytometry are especially noteworthy since these parameters (viability, acrosomal and mitochondrial status, and especially chromatin status) could provide important physiological information for this breed. The analysis of sperm viability is one of the most critical parameters in evaluating fertility in a particular male or the assessment of methods of semen preservation [47]. Cryopreservation leads to structural injuries in the sperm plasma and acrosomal membranes and causes functional damage [48]. Near 50% of pig, spermatozoa do not survive the freezing-thawing process [15,49]. In the samples assessed in this study, the post-thawing sperm viability showed promising results. Indeed, these results agree with those of Waterhouse et al. [46] using Norwegian Landrace and Duroc breeds and Yi et al. [50] with Yorkshire boars. Apoptosis is another indicator that could help assess the plasma membrane insult after sperm thawing, and it could be related to the activation of cellular pathways leading to death or decreased functionality [51]. In our study, apoptosis was low in the live sperm population, indicating low sub-lethal damage to the plasma membranes during the cryopreservation process and maybe better post-thawing performance if the doses were applied by artificial insemination. Similarly, acrosomal damage and ROS production were low, which might be a good indication of resilience to the cryopreservation process [52].

Besides, maintaining the mitochondrial integrity and functionality is critical to spermatozoa, from energy production to life-death signalling balance [53]. The boar spermatozoa rely on mitochondrial ATP for motility, and therefore live spermatozoa could have their function compromised if their mitochondrial membrane potential (MMP) is low [34]. Therefore, changes in mitochondrial membrane potential could be a good indicator of functional impairment [54]. However, the sperm samples assessed in this study showed that most live spermatozoa kept active mitochondria (high MMP). This may imply that the mitochondria integrity was conserved after the frozen-thawed process, with a potential effect on the maintenance of sperm motility.

Sperm chromatin assessment is a critical element of sperm evaluation due to its impact on sperm fertility and offspring health [55,56]. Although some studies showed that DNA fragmentation could be low in boar semen, even after thawing [57], some males could be more susceptible to DNA damage [36]. Didion et al. [58] stated that the SCSA is a robust and statistically rigorous diagnostic technique that can identify subfertile boars. The same authors suggested that boar semen samples with a %DFI higher than 6% may yield reduced fertility and prolificacy. Thus, our study suggests that the Gochu Asturcelta boars selected for the GBR-PA not only produce semen with high chromatin integrity but also resist cryopreservation and might have little susceptibility to further damage. Therefore, the chromatin status of spermatozoa would not affect the performance of these samples, following previous studies on this topic [58,59,60,61].

Considering the individual male variability, we found a significant boar effect affecting most of the seminal parameters during the study. These results agree with Roca et al. [15], which found that boar was the primary factor influencing the ejaculate variability in sperm cryo-survival. They suggested that this factor should be an essential criterion for selecting ejaculates for cryopreservation. Whereas sperm freezability might be a criterion for removing sub-optimal males in commercial settings, this is not an option for local endangered breeds when maintaining the breed’s viability and genetic diversity is a priority [62]. However, even though we detected this male effect, the overall results after thawing and even after the post-thawing incubation suggest an excellent performance for the assessed males and that the germplasm bank has high viability and potential for future use.

Interestingly, the boar’s age was not a relevant factor in our results, despite its wide range (8 to 71 months). The boar age effect is a controversial topic, maybe because the useful life of commercial boars is relatively short. Nevertheless, some effects have been reported, especially in very young and older animals [63,64,65]. Considering sperm cryopreservation, Joyal et al. [40] found that as the boar’s age increased, both total motile and progressive sperm declined post-thawing. However, Roca et al. [15] minimized the importance of age on post-thawing sperm quality. With a non-commercial breed, our study seems to follow these findings, although this is a complex topic that might require a dedicated and careful design.

The most relevant finding of the present study is detecting and characterization of a significant effect of the collection season. This is a controversial factor in commercial breeds, with some researchers reporting a significant variability along the year [66,67], including higher production of spermatozoa in autumn and winter and lower production in summer [63,68], mainly due to heat stress [69]. Other studies have disregarded a significant effect of the season when barn conditions are well controlled, whereas seasonality effects are more relevant in hot or tropical climates [69,70]. Also, Barranco et al. [25] showed that the spermatozoa from ejaculates collected during summer and, to a lesser extent, also in autumn, were more sensitive to cryopreservation than those from ejaculates collected during winter and spring. However, not all the research in this field is in agreement with the above studies. Other authors found that the sperm quality of the selected boars decreased during decreasing photoperiods compared with increasing photoperiods, mainly due to impaired testicular function [71], which could partly explain our results. Petrocelli et al. [72] found that semen quality was negatively affected during autumn and was related to photoperiod changes in boars living in temperate climates. Contrarily, Rivera et al. [73] stated that the Mediterranean photoperiod did not strongly affect overall boar-semen quality. Likewise, the breed should be considered when assessing the seasonality of sperm quality, as sperm motility of frozen-thawed sperm in Yorkshire boars was higher than in Duroc boars in spring and summer [74]. Our results with the Gochu Astucelta breed showed that season has some effect on sperm quality after thawing, with semen collecting by wintertime resulting in lower post-thawing quality than in other periods of the year and better results in most sperm parameters by spring-summer. Argenti et al. [75] indicated that boar semen productivity was affected by seasonality in a subtropical area. However, its effects were not equal among different regions, which researchers need to consider when interpreting the results of different studies. 

The controversy around the effect of season on semen and frozen-thawed sperm quality could be due to different factors such as location/climate where the study was performed, photoperiod, breed, environment, and research conditions that could affect the research outcome. For example, in some of these studies, high temperature during summer was considerable, which resulted in the heat stress of the boars. In this regard, the Gochu Asturcelta breed is extensively reared, therefore exposed to photoperiodic and climatic conditions. However, the area of Northern Spain where this native breed is raised and where this study took place has a temperate climate with mild summers and winters. The temperature oscillates between 7 °C and 23 °C in nearby Gijón (Asturias) during the year. In the summer, the average highest temperature is 23 °C in July–August, while in the winter, the average lowest temperature is 7 °C in December-January. The daylight lasts 15.5 h in summer (measure in June 21st) and almost 9 h in winter (measure in December 21st). Indeed, the meteorological dataset used in this study (data recorded from an observatory nearby) showed a maximum daily temperature of 22.2 °C in all the studied periods. Therefore, we hypothesize that these boars, both because of the climate and their rusticity, would be very well adapted to these environmental conditions and would not suffer from heat stress in the summer. The seasonality effect in our study was also greatly evidenced by the post-thawing incubation. This post-thawing incubation of the samples causes an overall decrease in sperm quality [22], something reported in our study and expected, but it often enhances minor differences between treatments [50,54,76,77]. This is very important since these differences in post-thawing resilience might result from hidden insults from the cryopreservation process, potentially developing and becoming significant during the application of the semen in assisted reproductive technologies.

The lower sperm freezability in the winter is intriguing, and a photoperiodic effect could cause it, somewhat following previous reports of lower sperm quality in decreasing photoperiod [71,72]. We tried to find out the source of these changes by combining actual meteorological data with our sperm quality results. As we hypothesized, the temperature was not a relevant factor in sperm quality, in line with the area’s mild climate, with problems such as heat stress in summer absent in that environment. More interesting is the consistent association of the photoperiod (daylight hours) with the pre-freezing sperm quality (again, mainly in the samples incubated for 150 min post-thawing). These findings suggest that the better results found in spring and summer could be due to longer days and maybe increasing photoperiod, in line with some studies [71,72]. We must remind that the meteorological and daylength data used in this study was not limited to the day of collection. However, it included seven weeks before the collection day, taking into account influences during the spermatogenesis and sperm maturation periods. Further research is warranted here to solve this potential problem on sperm preservation for this breed and because it could shed light on the effects of the environment on the physiology of autochthonous domestic breeds from different areas.

The number of boars used in this study was necessarily limited, but since the Gochu Asturcelta breed is at risk of extinction, this boar sample could represent the current population. This is the first report on frozen-thawed sperm quality of the pig breed Gochu Asturcelta. It confirms that the frozen sperm doses of this breed stored at the GRB of Principado de Asturias have an excellent preservation level, with further possibility to be used if necessary.

## 5. Conclusions

Our findings demonstrated that the post-thawing sperm quality of Gochu Asturcelta was in the range of results for commercial breeds, bringing a good prospect for the use of assisted reproductive technologies in this local breed. Despite being rescued from a reduced number of original pigs, the semen parameters, notably chromatin status, showed good condition. Remarkably, we detected seasonal effects on the seminal quality post-thawing that could condition sperm cryopreservation programs in this breed and might be relevant for cryobank management. In addition, the information obtained in this study could help the conservation of another native, non-commercial and endangered breeds.

## Figures and Tables

**Table 1:** Data distribution for the motility parameters (CASA) in cryopreserved spermatozoa from Gochu Asturcelta breed, post-thawing analysis.

Parameter	Lower Hinge	Q1	Median	Q3	Higher Hinge
Total motility (%)	18.0	34.0	44.0	51.0	70.0
Progressive motility (%)	12.0	28.0	36.0	45.0	64.0
VCL (µm/s)	50.5	66.8	74.3	84.7	106.3
VSL (µm/s)	22.8	40.2	47.9	52.8	71.0
VAP (µm/s)	39.8	51.4	56.6	64.5	82.8
LIN (%)	41.4	59.4	68.7	76.3	87.7
STR (%)	74.6	86.7	92.8	95.5	99.4
WOB (%)	59.9	71.4	80.5	84.5	89.7
ALH (µm)	1.6	2.1	2.3	2.7	3.5
BCF (Hz)	6.0	7.0	8.0	8.0	9.0
DNC (µm^2^/s)	77.8	137.6	169.8	205.8	304.3
DNCm (µm)	2.1	2.9	3.7	5.0	8.1

Q1 and Q3 are the first and third quartiles of the data distribution. The lower and upper hinge are the most extreme values within 1.5 times the interquartile range (minimum and maximum not considering extreme values—outliers). VCL, curvilinear velocity; VSL, straight-line velocity; VAP, average path velocity; LIN, linearity of the curvilinear trajectory (ratio of VSL/VCL); STR, straightness; ALH, amplitude of lateral head displacement; WOB, wobble coefficient; BCF, beat cross-frequency; DNC, sperm dance; DNCm, sperm mean dance.

**Table 2:** Data distribution for the physiological and chromatin parameters (flow cytometry) in cryopreserved spermatozoa from Gochu Asturcelta breed, post-thawing analysis.

Parameter	Lower Hinge	Q1	Median	Q3	Higher Hinge
Viability (%)	34.0	51.3	60.0	65.5	74.0
Damaged acrosomes (ratio viable) (%)	0.2	0.4	0.6	1.0	1.5
Damaged acrosomes (%)	9.2	13.5	18.4	21.4	32.3
Apoptotic sperm (ratio) (%)	1.1	2.7	4.9	8.7	17.0
Cytoplasmic ROS (ratio viable) (%)	4.3	11.4	19.6	34.0	58.5
Active mitochondria (ratio viable) (%)	59.9	75.1	87.0	93.9	98.0
SD-DFI	11.5	13.5	15.1	17.0	22.1
%DFI (%)	0.0	0.4	0.7	1.0	1.4
%HDS (%)	5.3	8.6	10.0	11.4	15.1

Q1 and Q3 are the first and third quartiles of the data distribution. The lower and upper hinge are the most extreme values within 1.5 times the interquartile range (minimum and maximum not considering extreme values—outliers). ROS, Reactive oxygen species; ROS, SD-DFI, standard deviation of the DNA fragmentation index (DFI); %DFI, proportion of spermatozoa with high DFI (damaged DNA); %HDS, proportion of spermatozoa with immature or decondensed chromatin.

**Table 3:** Data distribution for the motility parameters (CASA) in cryopreserved spermatozoa from Gochu Asturcelta breed after 150 min of incubation at 37 °C.

Parameter	Lower Hinge	Q1	Median	Q3	Higher Hinge
Total motility (%)	5.0	19.5	28.5	39.5	59.0
Progressive motility (%)	3.0	15.0	23.0	33.0	55.0
VCL (µm/s)	40.7	56.5	63.8	70.5	91.2
VSL (µm/s)	21.9	32.1	41.9	49.2	68.8
VAP (µm/s)	30.9	40.0	49.5	56.0	77.1
LIN (%)	50.6	61.4	71.7	78.7	84.2
STR (%)	81.9	89.4	92.0	95.4	99.2
WOB (%)	65.9	72.8	79.6	83.8	88.6
ALH (µm)	1.5	1.9	2.1	2.3	2.7
BCF (Hz)	5.8	7.0	7.8	8.0	9.0
DNC (µm^2^/s)	60.3	108.8	130.8	170.1	229.6
DNCm (µm)	2.1	2.7	3.3	3.9	5.1

Q1 and Q3 are the first and third quartiles of the data distribution. The lower and upper hinge are the most extreme values within 1.5 times the interquartile range (minimum and maximum not considering extreme values—outliers). VCL, curvilinear velocity; VSL, straight-line velocity; VAP, average path velocity; LIN, linearity of the curvilinear trajectory (ratio of VSL/VCL); STR, straightness; ALH, amplitude of lateral head displacement; WOB, wobble coefficient; BCF, beat cross-frequency; DNC, sperm dance; DNCm, sperm mean dance.

**Table 4:** Data distribution for the physiological and chromatin parameters (flow cytometry) in cryopreserved spermatozoa from Gochu Asturcelta breed after 150 min of incubation at 37 °C.

Parameter	Lower Hinge	Q1	Median	Q3	Higher Hinge
Viability (%)	30.6	44.4	53.3	59.2	68.6
Damaged acrosomes (ratio viable) (%)	0.0	1.0	1.7	2.6	4.4
Damaged acrosomes (%)	0.0	17.9	24.0	31.8	47.9
Apoptotic sperm (ratio) (%)	1.1	6.6	12.1	17.0	31.7
Cytoplasmic ROS (ratio viable) (%)	2.0	11.9	16.8	33.8	61.7
Active mitochondria (ratio viable) (%)	38.5	67.1	81.0	88.3	97.1
SD-DFI	11.9	14.3	15.9	17.6	21.6
%DFI (%)	0.1	0.3	0.6	0.8	1.4
%HDS (%)	6.7	10.6	12.0	13.6	16.0

Q1 and Q3 are the first and third quartiles of the data distribution. The lower and upper hinge are the most extreme values within 1.5 times the interquartile range (minimum and maximum not considering extreme values—outliers). ROS, Reactive oxygen species; SD-DFI, standard deviation of the DNA fragmentation index (DFI); %DFI, proportion of spermatozoa with high DFI (damaged DNA); %HDS, proportion of spermatozoa with immature or decondensed chromatin.

## Data Availability

The data presented in this study are available on request from the corresponding author.

## Supplementary Materials

The following are available online at https://www.mdpi.com/article/10.3390/ani11071885/s1, Figure S1: Gating strategy and representative cytograms and histograms for the flow cytometry analysis of frozen-thawed spermatozoa of the Gochu Asturcelta pig breed. Initially, a FSC/SSC region (**A**, P1) was draw enclosing events compatible with spermatozoa. A histogram on Hoechst 33342 fluorescence allowed (**B**) allowed identifying a high-fluorescence population with nucleated events (P2). A logical P1 and P2 gate was built and applied to the other plots. Viability and acrosomal integrity were assessed in a PNA-FITC/PI cytogram (**C**) with quadrants enclosing (Q1) dead sperm with undamaged acrosome (PI^+^/PNA-FITC^–^); (Q2): dead sperm with damaged acrosome (PI^+^/PNA-FITC^+^); (Q3) live sperm with non-reacted acrosome (PI^–^/PNA-FITC^–^); (Q4) live sperm with reacted acrosome (PI^–^/PNA-FITC^+^). Mitochondrial activity (**D**) was assessed in the live population by gating in the live population (histogram D-P2, region P3 of PI^–^ events) and analyzing the Mitotracker deep red fluorescence in a histogram (D-P3), taking as positive those events with high fluorescence (region P5). The production of cytoplasmic reactive oxygen species (**E**) was assessed in the live population by gating in the live population (histogram E-P2, region P3 of PI^–^ events) and analyzing the CM-H_2_DCFDA fluorescence in a histogram (E-P3), taking as positive those events with high fluorescence (region P4). The occurrence of apoptotic-like events (**F**) was assessed in the live population by gating in the live population (histogram F-P2, region P3 of PI^–^ events) and analyzing the YO PRO 1 fluorescence in a histogram (F-P3), taking as positive ¬those events with high fluorescence (region P5). File S1: *p* values from multiple comparison tests, File S2: *p*-values from the fixed part of the models for assessing the effects of meteorological parameters and daylength
