# Supplementary material for: Post-Thaw Sperm Quality and Functionality in the Autochthonous Pig Breed Gochu Asturcelta
Source: Animals (Basel). 2021 Jun 24;11(7):1885. doi: 10.3390/ani11071885 (PMC8300257; doi:10.3390/ani11071885)
Supplement: Supplementary file 1 [file animals-11-01885-s001.zip › animals-1238554-supplementary.pdf]

animals

MDPI

Post-thaw sperm quality and functionality in the autochthonous pig breed Gochu Asturcelta

José Néstor Caamaño, Carolina Tamargo, Inmaculada Parrilla, Felipe Martínez-Pastor, Lorena Padilla, Amer Salman, Carmen Fueyo, Ángel Fernández, María José Merino, Tania Iglesias and Carlos Olegario Hidalgo

Supplementary material

1. Cytograms and gating strategy for the flow cytometry analysis

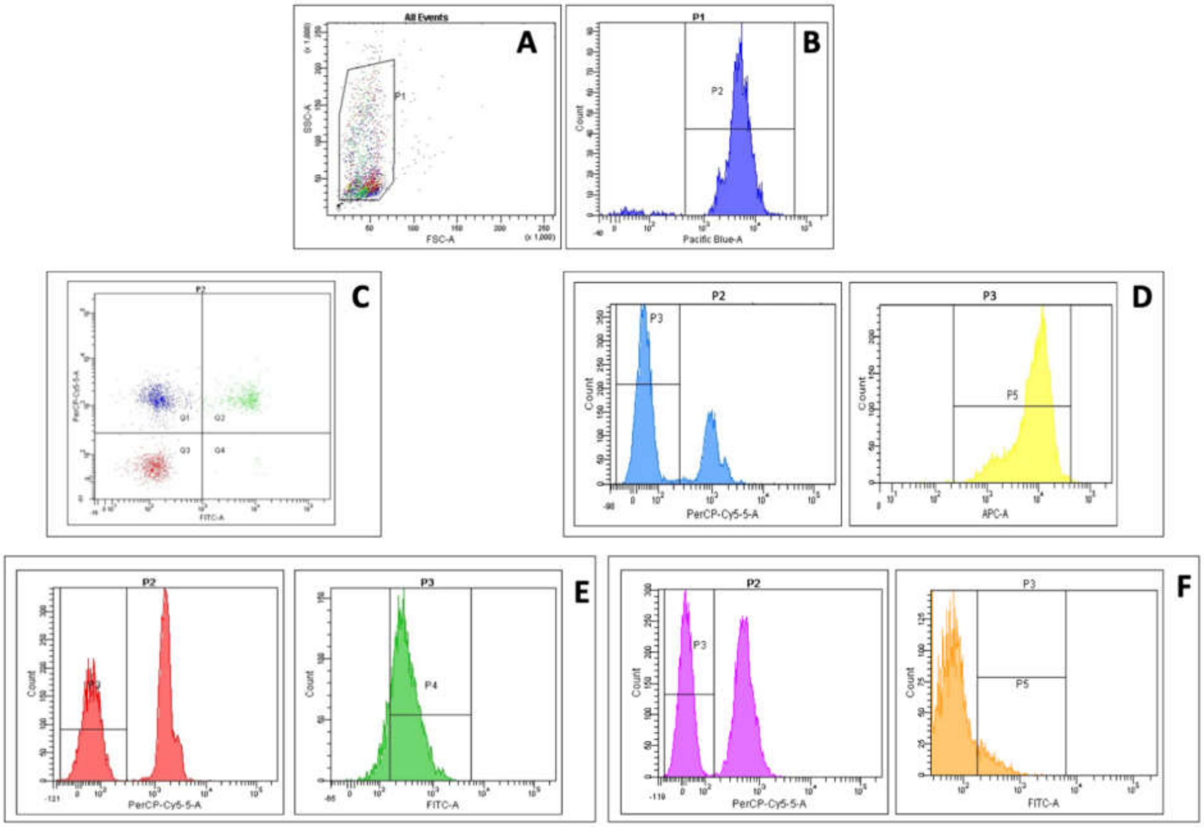

Figure S1. Gating strategy and representative cytograms and histograms for the flow cytometry analysis of frozen-thawed spermatozoa of the Gochu Asturcelta pig breed. Initially, a FSC/SSC region (A, P1) was draw enclosing events compatible with spermatozoa. A histogram on Hoechst 33342 fluorescence allowed (B) allowed identifying a high-fluorescence population with nucleated events (P2). A logical P1 and P2 gate was built and applied to the other plots. Viability and acrosomal integrity were assessed in a PNA-FITC/PI cytogram (C) with quadrants enclosing (Q1) dead sperm with undamaged acrosome (PI^{+}/$PNA\text{-}FITC^{-}$); (Q2): dead sperm with damaged acrosome (PI^{+}/$PNA\text{-}FITC^{+}$); (Q3) live sperm with non-reacted acrosome (PI^{-} /$PNA\text{-}FITC^{-}$); (Q4) live sperm with reacted acrosome (PI^{-}/$PNA\text{-}FITC^{+}$). Mitochondrial activity (D) was assessed in the live population by gating in the live population (histogram D-P2, region P3 of PI^{-} events) and analyzing the Mitotracker deep red fluorescence in a histogram (D-P3), taking as positive those events with high fluorescence (region P5). The production of cytoplasmic reactive oxygen species (E) was assessed in the live population by gating in the live population (histogram E-P2, region P3 of PI^{-} events) and analyzing the CM-H_2DCFDA fluorescence in a histogram (E-P3), taking as positive those events with high fluorescence (region P4). The occurrence of apoptotic-like events (F) was assessed in the live population by gating in the live population (histogram F-P2, region P3 of PI^{-} events) and analyzing the YO-PRO-1 fluorescence in a histogram (F-P3), taking as positive those events with high fluorescence (region P5).

File S1. P values from multiple comparison tests

The figures in the main manuscript shows significant differences between seasons by using different letters, following the convention that groups not sharing letters differ P<0.05. The following tables detail the results of the multiple comparison (contrasts with Tukey correction) wherever the season was significant.

Initial motility (fresh semen)

contrast	estimate	SE	df	t.ratio	p.value
Spring - Summer	-0.11053	0.0358	35.6	-3.089	0.0233
Spring - Fall	-0.10216	0.0467	49.0	-2.186	0.1346
Spring - Winter	-0.15946	0.0618	42.9	-2.581	0.0668
Summer - Fall	0.00836	0.0493	47.2	0.170	0.9057
Summer - Winter	-0.04894	0.0645	46.8	-0.759	0.9057
Fall - Winter	-0.05730	0.0549	47.8	-1.044	0.9057

Total motility 150 min

contrast	estimate	SE	df	t.ratio	p.value
Spring - Summer	0.0438	0.0329	33.9	1.333	0.3829
Spring - Fall	0.0928	0.0446	45.5	2.079	0.1300
Spring - Winter	0.2770	0.0624	45.6	4.441	0.0003
Summer - Fall	0.0490	0.0464	42.3	1.056	0.3829
Summer - Winter	0.2332	0.0644	46.9	3.623	0.0036
Fall - Winter	0.1843	0.0544	44.6	3.384	0.0060

Progressive motility 150 min

contrast	estimate	SE	df	t.ratio	p.value
Spring - Summer	0.0393	0.0314	34.0	1.251	0.4393
Spring - Fall	0.0923	0.0424	45.6	2.174	0.1047
Spring - Winter	0.2649	0.0590	45.2	4.487	0.0003
Summer - Fall	0.0530	0.0442	42.6	1.199	0.4393
Summer - Winter	0.2256	0.0610	46.8	3.697	0.0029
Fall - Winter	0.1727	0.0518	44.9	3.334	0.0069

VSL 30 min

contrast	estimate	SE	df	t.ratio	p.value
Spring - Summer	0.0293	0.0616	36.1	0.475	0.6377
Spring - Fall	0.1583	0.0765	47.7	2.070	0.1756
Spring - Winter	0.3004	0.0964	35.5	3.117	0.0217
Summer - Fall	0.1291	0.0820	47.4	1.575	0.3658
Summer - Winter	0.2711	0.1022	41.1	2.652	0.0566
Fall - Winter	0.1421	0.0907	47.8	1.566	0.3658

VSL 150 min

contrast	estimate	SE	df	t.ratio	p.value
Spring - Summer	0.0157	0.0577	33.7	0.272	0.7874
Spring - Fall	0.2692	0.0791	45.2	3.401	0.0085
Spring - Winter	0.1740	0.1115	46.1	1.560	0.5023
Summer - Fall	0.2535	0.0819	41.8	3.094	0.0176
Summer - Winter	0.1583	0.1148	47.0	1.380	0.5228
Fall - Winter	-0.0952	0.0964	44.0	-0.987	0.6579

LIN 150 min

contrast	estimate	SE	df	t.ratio	p.value
Spring - Summer	-0.0128	0.0212	35.6	-0.605	0.5492
Spring - Fall	0.0644	0.0281	46.3	2.294	0.0528
Spring - Winter	0.1733	0.0384	44.0	4.509	0.0002
Summer - Fall	0.0773	0.0294	43.9	2.626	0.0356
Summer - Winter	0.1861	0.0400	46.1	4.659	0.0002
Fall - Winter	0.1088	0.0343	46.0	3.170	0.0109

STR 150 min

contrast	estimate	SE	df	t.ratio	p.value
Spring - Summer	-0.0267	0.0240	33.7	-1.115	0.5453
Spring - Fall	0.0325	0.0298	47.0	1.088	0.5453
Spring - Winter	0.0898	0.0386	34.6	2.325	0.1303
Summer - Fall	0.0592	0.0319	45.5	1.859	0.2781
Summer - Winter	0.1165	0.0408	39.9	2.854	0.0409
Fall - Winter	0.0573	0.0365	47.0	1.568	0.3704

WOB 150 min

contrast	estimate	SE	df	t.ratio	p.value
Spring - Summer	0.00458	0.0181	35.5	0.253	0.8018
Spring - Fall	0.04378	0.0240	46.2	1.822	0.2248
Spring - Winter	0.11341	0.0329	44.1	3.446	0.0076
Summer - Fall	0.03919	0.0252	43.8	1.558	0.2530
Summer - Winter	0.10883	0.0342	46.2	3.182	0.0131
Fall - Winter	0.06963	0.0294	45.9	2.371	0.0879

BCF 150 min

contrast	estimate	SE	df	t.ratio	p.value
Spring - Summer	0.0357	0.0316	35.8	1.128	0.5335
Spring - Fall	0.0548	0.0386	46.9	1.419	0.4878
Spring - Winter	0.1431	0.0492	34.0	2.910	0.0380
Summer - Fall	0.0192	0.0415	46.3	0.462	0.6460
Summer - Winter	0.1075	0.0523	39.0	2.056	0.2324
Fall - Winter	0.0883	0.0473	46.9	1.868	0.2723

DNC mean 150 min

contrast	estimate	SE	df	t.ratio	p.value
Spring - Summer	0.0255	0.0484	35.9	0.526	1.0000
Spring - Fall	-0.0369	0.0620	46.9	-0.596	1.0000
Spring - Winter	-0.2247	0.0822	40.5	-2.733	0.0463
Summer - Fall	-0.0624	0.0656	45.1	-0.951	1.0000
Summer - Winter	-0.2502	0.0863	44.0	-2.900	0.0349
Fall - Winter	-0.1878	0.0758	46.8	-2.478	0.0675

Viability 30 min

contrast	estimate	SE	df	t.ratio	p.value
Spring - Summer	-0.0440	0.0213	40.7	-2.071	0.0895
Spring - Fall	0.0395	0.0253	47.3	1.564	0.1244
Spring - Winter	0.1745	0.0315	36.3	5.548	<.0001
Summer - Fall	0.0836	0.0274	49.0	3.047	0.0111
Summer - Winter	0.2186	0.0337	41.1	6.493	<.0001
Fall - Winter	0.1350	0.0304	48.9	4.447	0.0002

Viability 150 min

contrast	estimate	SE	df	t.ratio	p.value
Spring - Summer	-0.0173	0.0216	40.4	-0.803	0.4266
Spring - Fall	0.0401	0.0265	48.3	1.514	0.2732
Spring - Winter	0.1571	0.0337	39.5	4.666	0.0002
Summer - Fall	0.0575	0.0285	48.9	2.017	0.1477
Summer - Winter	0.1745	0.0357	43.8	4.881	0.0001
Fall - Winter	0.1170	0.0316	49.0	3.701	0.0022

Acrosomal damage in viable 150 min

contrast	estimate	SE	df	t.ratio	p.value
Spring - Summer	0.004111	0.0189	35.5	0.217	1.0000
Spring - Fall	0.004318	0.0233	47.9	0.186	1.0000
Spring - Winter	0.090670	0.0296	34.5	3.064	0.0211
Summer - Fall	0.000206	0.0250	48.8	0.008	1.0000
Summer - Winter	0.086559	0.0314	40.7	2.757	0.0348
Fall - Winter	0.086352	0.0277	49.0	3.113	0.0185

Mitochondrial activity ratio 150 min

contrast	estimate	SE	df	t.ratio	p.value
Spring - Summer	0.000372	0.0472	39.0	0.008	0.9937
Spring - Fall	0.160073	0.0596	48.8	2.684	0.0396
Spring - Winter	0.231700	0.0772	41.4	3.001	0.0273
Summer - Fall	0.159700	0.0636	48.4	2.512	0.0461
Summer - Winter	0.231328	0.0813	45.5	2.844	0.0332
Fall - Winter	0.071628	0.0706	48.8	1.014	0.6310

File S2. P-values from the fixed part of the models for assesing the effects of meteorological parameters and daylength

The figures in the main manuscript shows significant differences between seasons by using different letters, following the convention that groups not sharing letters differ P<0.05. The following tables detail the results of the multiple comparison (contrasts with Tukey correction) wherever the season was significant.

Pre-freezing

Variable	Mean Temperature	Precipitation	Daylength
Concentration	0.004	0.099	0.757
Motility, fresh	0.004	0.526	0.062
Motility, pre-freezing	0.908	0.218	0.315

Post-thawing, 30 min incubation

Variable	Mean Temperature	Precipitation	Daylength
Total motility	0.271	0.413	0.622
Progressive motility	0.374	0.736	0.763
VCL	0.721	0.58	0.033
VSL	0.91	0.331	0.063
VAP	0.842	0.824	0.053
LIN	0.489	0.398	0.479
STR	0.894	0.554	0.29
WOB	0.593	0.272	0.753
ALH	0.585	0.404	0.406
BCF	0.653	0.453	0.668
DNC	0.69	0.671	0.194
DNCm	0.385	0.32	0.773
Viability	0.103	0.341	0.001
Acrosomal damage, viable	0.985	0.23	0.997
Acrosomal damage, total	0.683	0.468	0.641
Apoptotic ratio	0.271	0.293	0.62
ROS ratio	0.724	0.511	0.101
Mitochondrial activity ratio	0.475	0.912	0.051
SD-DFI	0.574	0.597	0.602
%DFI	0.157	0.401	0.184
%HDS	0.725	0.584	0.588

Post-thawing, 150 min incubation

Variable	Mean Temperature	Precipitation	Daylength
Total motility	0.563	0.559	0.088
Progressive motility	0.632	0.48	0.058
VCL	0.466	0.744	0.065
VSL	0.064	0.91	0.005
VAP	0.18	0.589	0.044
LIN	0.635	0.179	0.002
STR	0.428	0.444	0.027
WOB	0.779	0.257	0.031
ALH	0.781	0.316	0.993
BCF	0.977	0.651	0.244
DNC	0.347	0.458	0.227
DNCm	0.367	0.105	0.086
Viability	0.235	0.514	0.013
Acrosomal damage, viable	0.2	0.373	0.684
Acrosomal damage, total	0.735	0.7	0.54
Apoptotic ratio	0.501	0.876	0.294
ROS ratio	0.395	0.419	0.075
Mitochondrial activity ratio	0.371	0.983	0.005
SD-DFI	0.355	0.333	0.482
%DFI	0.534	0.578	0.964
%HDS	0.198	0.124	0.003
